# Frequency Instability Impact of Low-Cost SDRs on Doppler-Based Localization Accuracy

**DOI:** 10.3390/s24041053

**Published:** 2024-02-06

**Authors:** Kacper Bednarz, Jarosław Wojtuń, Jan M. Kelner, Krzysztof Różyc

**Affiliations:** Institute of Communications Systems, Faculty of Electronics, Military University of Technology, 00-908 Warsaw, Poland; kacper.bednarz@wat.edu.pl (K.B.); krzysztof.rozyc@wat.edu.pl (K.R.)

**Keywords:** signal Doppler frequency (SDF), frequency stability, software-defined radio (SDR), localization, wireless communication

## Abstract

In this paper, we explore several widely available software-defined radio (SDR) platforms that could be used for locating with the signal Doppler frequency (SDF) method. In the SDF, location error is closely related to the accuracy of determining the Doppler frequency shift. Therefore, ensuring high frequency stability of the SDR, which is utilized in the location sensor, plays a crucial role. So, we define three device classes based on the measured frequency stability of selected SDRs without and with an external rubidium clock. We estimate the localization accuracy for these classes for two scenarios, i.e., short- and long-range. Using an external frequency standard reduces the location error from 20 km to 30 m or 15 km to 2 m for long- and short-range scenarios, respectively. The obtained simulation results allowed us to choose an SDR with appropriate stability. The studies showed that using an external frequency standard is necessary for minimizing SDR frequency instability in the Doppler effect-based location sensor. Additionally, we review small-size frequency oscillators. For further research, we propose two location sensor systems with small size and weight, low power consumption, and appropriate frequency stability. In our opinion, the SDF location sensor should be based on the bladeRF 2.0 micro xA4 or USRP B200mini-i SDR platform, both with the chip-scale atomic clock CSAC SA.45s, which will allow for minor positioning errors in the radio emitters.

## 1. Introduction

The dynamic growth of the microelectronics market at the turn of the 20th and 21st centuries had a significant impact on the progress of many branches of industry and science, including mobile telephony [1] and unmanned platforms, i.e., unmanned ariel vehicles (UAVs) [2,3]. The advancement of the mobile network contributed to the creation of software-defined radio (SDR) technology [4]. Currently, most modern radio communication devices are based on SDR technology. In 2022, the global SDR market value was estimated at USD 21 billion. In 2028, this value is forecast to reach USD 32.2 billion (an increase of 53.3% compared to 2022) at a compound annual growth rate (CAGR) equal to 7.4% [5]. Forecasts regarding the development of the UAV market are even more optimistic. In 2023, the value of the global UAV market (including fixed-wing, hybrid, and multi-rotor drones) and its applications (including agriculture, audit, surveillance, inspection and monitoring, consumer goods, and retail) is estimated at USD 44.57 billion. Forecasts indicate that in 2030 this market will increase unimaginably, by 1684.9% compared to 2023, reaching USD 795.57 billion [6] at a CAGR equal to 50.9%. These growth dynamics contribute to the search for new applications for SDR and UAV technologies.

This paper focuses on the frequency instability of SDR platforms and their potential use on UAVs in electronic warfare (EW) applications. Based on the measured frequency stability parameters of selected SDRs, we estimate the position errors of radio emitters using the Doppler-based localization method and a UAV with an SDR receiver. The aim of the research is to select an appropriate SDR platform for the localization application. Wider context on this topic is provided in Section 2.

## 2. Related Works

Although the idea of SDR dates back to the 1970s, it was only the rapid development of digital electronics that allowed the practical implementation of many elements that had previously been considered theoretically. It is difficult to pinpoint the specific date of the invention of SDR, but many consider the system created in 1982 by Ulrich L. Rohde’s department, which used the complementary symmetry monolithic array computer chip (COSMAC), to be the first SDR. The 1984 SDR created by the Garland team was a digital baseband receiver that provided programmable noise cancellation and demodulation of broadband signals [7,8]. One of the key moments in the history of SDR was the work of Joseph Mitola III. In the 1980s and 1990s, Mitola published several articles and research papers in which he presented the concept of programmable radio, which was an important step towards the development of SDR. He heralded a decade of transition from hardware-based radios to software-intensive approaches [9,10]. Moreover, in the 1990s, field-programmable gate array (FPGA) technology was developed, which allowed for more effective implementation of algorithms and radio functions in the form of software. These developments have resulted in SDRs emerging in markets such as signals intelligence (SIGINT), EW, test and measurement, public safety communications, spectrum monitoring, and military communications (MILCOMs). The requirement for portability of SDR waveforms across hardware platforms has led to the development of tools such as the Software Communications Architecture (SCA) Core Framework, as well as better development tools from electronic design automation (EDA) and semiconductor companies. The future development of SDR technology is directly related to the development of technologies such as 5G, 6G, Internet of Things (IoT), and sensor networks. It is assumed that the next step in SDR hardware development will be to combine analog and digital technologies in one monolithic chip, reducing cost, size, weight, and power (SWaP). The development of the next generation of SDRs will involve the integration of analog and digital technologies with mixed-signal chips. At the moment, however, the limiting element is the software, not the hardware. Comprehensive use of next-generation SDRs will require development environments that can seamlessly program both general-purpose processors (GPPs) and FPGAs [4,11].

The UAV market should be considered primarily in two categories: platforms and applications. There are many works in the literature devoted to the classification of these two categories, e.g., [12,13,14,15]. On the other hand, the UAV market can be divided into the widely available civilian and military [16,17,18,19]. The military market, although much smaller (USD 12 billion in 2022, forecast to be USD 17 billion in 2027, a 7.3% CAGR [20]; or USD 15.88 billion in 2023, forecast to be USD 20.64 billion in 2027, a 6.8% CAGR [21]) than the civilian one, is specific due to dedicated platforms and applications that usually have no equivalents on the civilian market. On the other hand, with the advent of 5G, 6G, and beyond mobile networks, the development of the potential of non-terrestrial networks (NTNs) [22,23] makes it possible to secure military communications in difficult conditions [24,25].

An unmanned surveillance and reconnaissance aerial vehicle (USRAV) is an unarmed military UAV that realizes intelligence, surveillance, target acquisition, and reconnaissance (ISTAR) tasks [26]. Most USRAVs used by armies perform imagery intelligence (IMINT) tasks. For this purpose, cameras operating in various ranges of the optical spectrum are used, primarily in the visible (VIS) range but also in the infrared (IR) and ultraviolet (UV) ranges. In addition, synthetic-aperture radar (SAR) or light detection and ranging (LIDAR) technologies are used. This reconnaissance area is also used in many civilian applications. USRAVs performing signals SIGINT tasks are much less common. In the area of SIGINT, communication intelligence (COMINT) or electronic intelligence (ELINT) can be realized in the field of detection, recognition, and localization of telecommunications (including military communication systems) or non-communications emitters (i.e., radars), respectively. There are SIGINT systems mounted on UAVs available on the market. The leading manufacturers of this type of solution include:

- The United States—Northrop Grumman, West Falls Church, VA (RQ-4 Global Hawk, MQ-4C Triton), General Atomics, San Diego, CA (MQ-9 Reaper, Predator ER, Avenger), Boeing, Arlington, VA (EA-18G Growler), and Kratos Defense & Security Solutions, San Diego, CA (XQ-58 Valkyrie);
- Israel—Elbit Systems, Haifa (Hermes 450, Hermes 900) and Israel Aerospace Industries (IAI), Lod (Eitan, Heron);
- Italy—Leonardo-Finmeccanica, Rome (Selex ES Falco);
- Germany—EMT, Penzberg (Luna X-2000);
- Türkiye—Turkish Aerospace Industries (TAI), Ankara (Gözcü) and Baykar, Istanbul (Bayraktar TB2 with SIGINT system BSI-101);
- Russia—Kronstadt Group, Moscow (Orion).

Reconnaissance and EW systems are systems in the field of military intelligence and counteraction. Therefore, some countries prefer to develop and implement national solutions. In Poland, national SIGINT systems are used on manned ground and flying platforms. In the era of UAV advance, it is planned to develop and implement SIGINT systems on unmanned platforms. Such an attempt is being made as part of the project on “Command and control of group of COMINT radio-electronic reconnaissance unmanned aerial vehicles based on modern IT technologies”, acronym UAV-COMINT, financed by the National Center for Research and Development and implemented by the Military University of Technology and the MSP InnTech Sp. z o. o. company. Under this grant, UAVs carrying out COMINT tasks in the field of spectrum monitoring and locating radio emitters will be developed. For the second task, we plan to use a Doppler-based localization method called the signal Doppler frequency (SDF) [27,28]. On the other hand, the localization of radio emitters is widely used in the civil market, including, among others, in positioning wireless network users or users who illegally use licensed frequency bands. In the first case, techniques dedicated to mobile and WiFi networks are utilized. Whereas, regulatory authorities mainly use the second approach. So far, direction-finding methods are generally used for this purpose. In the future, using the SDF method on UAVs may be a good alternative for this type of application. It is also worth highlighting that the impact assessment of SDR instability on using these platforms in practice has a much broader context than locating emitters.

The frequency stability of the signal source and receiver plays a crucial role in location methods based on measuring the instantaneous frequency of the received signal. In the case of intra-system localization (e.g., in mobile networks), this problem can be easily solved. In the case of military SIGINT systems, the sensor does not affect the frequency stability of the located emitter. However, it is possible to ensure the appropriate stability of the receiver, which is an element of the location sensor. This paper focuses on the impact evaluation of the receiver’s frequency stability on the SDF localization accuracy based on simulation studies. The presented analysis is based on the results of frequency stability measurements of low-cost SDR platforms made without or with the use of a rubidium frequency standard. The obtained results show a significant influence of the receiver clock stabilization on the localization accuracy using the SDF method. The conducted research allows for the selection of a low-budget SDR and a frequency standard that will ultimately be used in the location sensor in the UAV-COMINT project.

This method of classifying SDR platforms in terms of use in the SDF-based location sensor determines the originality and innovation of the developed solution. The main contributions of this article are listed below.

1. We present the concept of a location sensor dedicated to a UAV application.
2. We introduce a review of SDR platforms in terms of the possibility of using them to build a location sensor, which is characterized by appropriate frequency stability, weight, and size, limited by the capabilities of the UAV payload.
3. Based on empirical studies, we classify SDR platforms regarding frequency stability.
4. We conduct simulation studies to assess how SDR platforms’ frequency stability affects the SDF location errors.
5. We present an overview of small-size frequency oscillators in terms of the possibility of using them to build a size-limited location sensor.
6. We propose a hardware structure of a location sensor with an appropriate frequency stability, small weight and dimensions, and low power consumption.

The remainder of the paper is organized as follows. Section 3 briefly discusses the SDF method. The results of measuring the frequency stability of low-cost SDR platforms are included in Section 4. In Section 5, assumptions, scenarios, and simulation study results are shown. The synthesis of the obtained results, which allows for the selection of an appropriate SDR and frequency standard for the UAV-COMINT location sensor, is described in Section 6. Section 7 provides a summary.

## 3. Signal Doppler Frequency Location Method

### 3.1. Emitter Positioning

Position estimation of the radio emitter in the SDF method is based on the Doppler frequency shift (DFS) measurement in the received signal. Movement of the transmitter or receiver is necessary for the Doppler effect to occur. This phenomenon in the location procedure is more straightforward when the measuring receiver (i.e., localization sensor) moves and the transmitter (i.e., localized emitter) is fixed.

The SDF method is based on the analytical description of the Doppler effect, in which the DFS is defined as a function of time and the position coordinates of the transmitter relative to the receiver [29]:

(1) $$f_{D}\left(t\right)=f_{D\max}\frac{x_{0}-vt}{\sqrt{{\left(x_{0}-vt\right)}^{2}+y_{0}^{2}+z_{0}^{2}}},$$

where $f_{D\max}=f_{0}v/c$ is the maximum DFS, $f_{0}$ is the carrier frequency of the transmitted signal, v and c are the receiver velocity and lightspeed, respectively, and $\left(x_{0},y_{0},z_{0}\right)$ is the actual emitter position relative to the sensor (receiver).

By transforming Equation (1) and measuring the DFSs at several intervals (at least for two moments in time, $t_{1}\;\mathrm{and}\;t_{2}$), we can estimate the emitter position $\left(\tilde{x},\tilde{y},\tilde{z}\right)$ relative to the sensor [30,31]:

(2) $$\{\begin{array}{l} \tilde{x}=v\frac{t_{1}p\left(t_{1}\right)-t_{2}p\left(t_{2}\right)}{p\left(t_{1}\right)-p\left(t_{2}\right)},\\ \tilde{y}=\pm \sqrt{{\left[v\frac{\left(t_{1}-t_{2}\right)p\left(t_{1}\right)p\left(t_{2}\right)}{p\left(t_{1}\right)-p\left(t_{2}\right)}\right]}^{2}-z_{0}^{2}}, \end{array}$$

where $p\left(t\right)=\sqrt{f_{D\max}/{\tilde{f}}_{D}\left(t\right)-1},$ ${\tilde{f}}_{D}\left(t\right)$ is the DFS estimated based on measurements, and $t_{1}\;\mathrm{and}\;t_{2}$ are two moments in time.

Equation (2) illustrates a simplified two-dimensional (2D) version of SDF which assumes that the sensor moves at a constant speed along a specific direction OX, i.e., v=(v,0,0)=const., and at a specific height, i.e., $\tilde{z}=z_{0}=\mathrm{const}.$ Three-dimensional (3D) SDF is presented in [27]. In this case, the sensor can move in different directions with variable speed. For the simulation studies presented in this paper, we used a simplified 2D SDF.

### 3.2. SDF Sensor Concept

Measurements [30] have shown that using ground vehicles introduces limitations to the localization procedure, including difficulties in maintaining a constant speed, changing the sensor motion direction, and multipath propagation resulting from the neighborhood of terrain obstacles [32]. The use of UAVs [27,28] or watercrafts [33] for this purpose provides greater opportunities, provides greater movement freedom, and improves propagation conditions by minimizing the impact of unfavorable phenomena.

In the ongoing UAV-COMINT project, the SDF-based localization sensor will be mounted on a UAV. In this case, considering the limitations of the used platform is crucial from the viewpoint of conducting reconnaissance operations and the location procedure. When designing the sensor, its dimensions, weight, power supply, and data exchange interfaces between the sensor and the UAV subsystem used to communicate with the ground operator station should primarily be considered. These sensor parameters must be appropriate from the UAV viewpoint, especially its payload and available cargo space, power supply, and communication capabilities.

**Figure 1:**
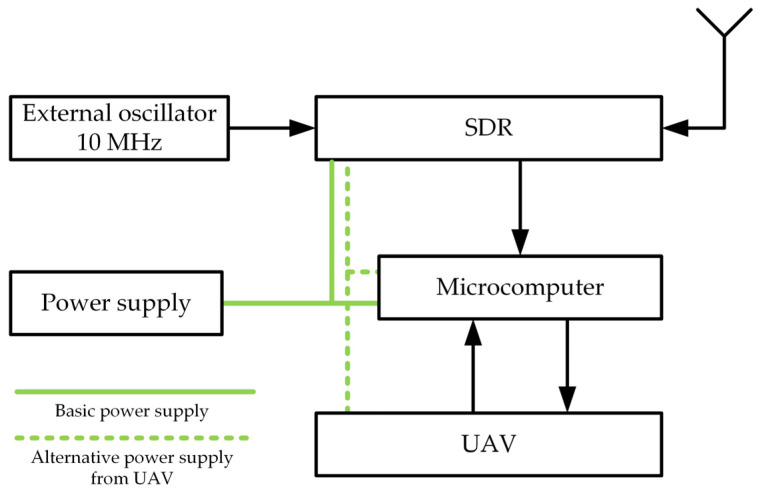
Conception of SDF sensor structure (green solid line – basic power supplying, green dashed line – alternative power supplying from UAV).

Figure 1 shows the structure and basic components of the SDF sensor, i.e., the microcomputer, SDR as a radio frequency (RF) receiver, and the receiving antenna.

In the Doppler-based location methods, frequency stability is essential for positioning accuracy. Measurements [31] showed that using an external frequency standard can significantly improve the accuracy of the SDF method. Therefore, when choosing an SDR, we additionally considered this parameter. For this purpose, the measurement of the stability of the SDR platforms was performed without and with a connected frequency standard, i.e., internal or external clock, respectively. Based on the conducted tests [34], an outline of which is presented in Section 4, we define representative parameter values for three classes of SDR devices, one without (i.e., with an internal clock) and two with an external clock, respectively. The simulation studies shown in Section 5 are based on these parameters. In these studies, we estimated the localization error, defined as

(3) $$\Delta r(t)=\sqrt{{\left(\tilde{x}(t)-x_{0}\right)}^{2}+{\left(\tilde{y}(t)-y_{0}\right)}^{2}}.$$

A summary of the research is presented in Section 6. Based on the simulation results obtained for two scenarios, we selected an SDR that we plan to use in the SDF sensor mounted on a UAV. Additionally, we plan to use a small external oscillator (see Figure 1) to improve localization accuracy. Therefore, we present a short overview of this type of device in the final part.

## 4. Frequency Stability of Low-Cost SDR Platforms

The frequency standard, or frequency oscillator, is a device that produces a periodic signal. When we speak about these devices, we think about the signals they generate and recognize that they have some nominal frequency. The term ‘frequency stability’ is used to characterize how small the frequency fluctuations of the oscillator signal are.

One of the definitions of ‘frequency instability’ is “the spontaneous and/or environmentally caused frequency change within a given time interval” [35,36,37]. The parameter frequency stability is often used when comparing one oscillator with another. In practice, when we use ‘frequency stability’ we mean ‘frequency instability’. The frequency stability does not determine whether the signal frequency is good or bad. It only indicates whether the frequency remains the same. It is important to note that the frequency of the signal produced by the oscillator can change over time. Some devices have good short-term stability and others have good long-term stability.

There are many statistics used to estimate frequency stability. One of the most common metrics is the Allan deviation [38]. Another parameter determining frequency stability is a dimensionless quantity defined as the ratio of frequency fluctuations to the nominal frequency. This metric is often called the fractional or normalized frequency fluctuation [36,37]. In this paper, we use this measure to determine the frequency stability [31,34] of selected SDR platforms.

**Figure 2:**
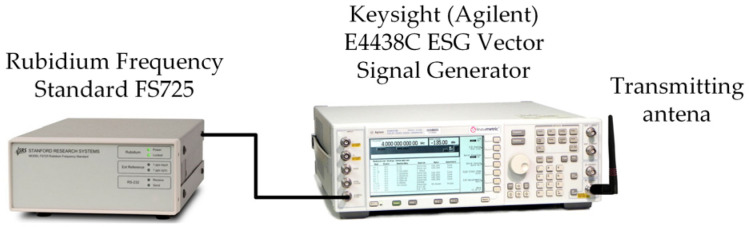
Transmitting part of test-bed for frequency stability measurement.

**Figure 3:**
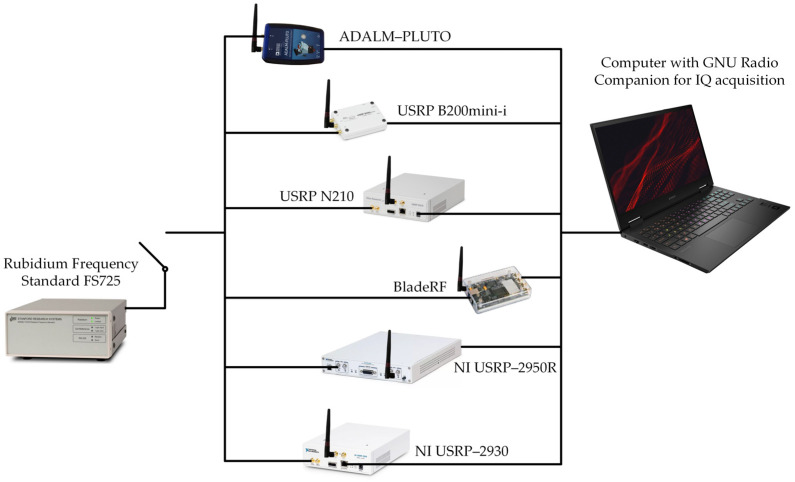
Receiving part of test-bed for frequency stability measurement.

The transmitting and receiving sides of the frequency stability measurement test-bed are illustrated in Figure 2 and Figure 3, respectively. The laboratory tests aim to determine the short-term frequency stability of the selected SDRs. The obtained frequency stability applies to the system consisting of a transmitting and receiving part. The measurement procedure is described in detail in [39].

A Keysight (Agilent), Santa Rosa, CA, E4438C ESG Vector Signal Generator with Rubidium Frequency Standard FS725 is the transmitting part of the test-bed. On the receiving side, we test six SDR platforms [40] with and without a 10 MHz reference clock from FS725:

- ADALM-PLUTO [41];
- Universal Software Radio Peripheral (USRP) B200mini-i [42];
- USRP N210 [43] with WBX, RFX1200 or XCVR2450 daughterboard [44,45];
- bladeRF 2.0 micro xA4 [46];
- USRP–2950R [47];
- USRP–2930 [48].

Due to our national regulations [49] and planned future empirical studies in a real environment, the Keysight generator is a source of a harmonic signal at carrier with a frequency $f_{0}=1358.01\;\mathrm{MHz}$. Using the GNU Radio Companion software ver. 3.10.5.0 and selected SDR, the IQ samples (i.e., in-phase and quadrature components) of the signal are recorded with bandwidth *B* = 200 kHz at a frequency f=1358 MHz. The 10 kHz offset between the generator and SDR allows us to ignore problems with IQ imbalance and direct current (DC) offset. After shifting the spectrum to a lower frequency range and considering a constant offset between the frequencies of the transmitted and received signals $\Delta f=f_{0}-f=10\;\mathrm{kHz},$ the instantaneous frequency of the received signal in the baseband should be $f_{b}=0\;\mathrm{kHz}$. Due to the instability of frequency oscillators, the instantaneous signal frequency changes over time. We determined the mean value $\mu_{f}$ and standard deviation $\sigma_{f}$ of the instantaneous frequency. To compare the instability of the SDR platforms, we determined the frequency stability, defined as follows [31,34]:

(4) $$s_{f}=\sigma_{f}/f_{0},$$

where $\sigma_{f}$ and $f_{0}$ are the standard deviation of the instantaneous frequency and the carrier frequency, respectively.

The frequency stability results measured for the analyzed SDRs are summarized in Table 1.

Based on the stability measurements, we propose grouping the SDR platforms into three classes. The first class, with the least stability, corresponds to devices that do not use an additional reference signal from an external frequency standard. For this class, we recommend using the frequency stability parameter with a representative value equal to $s_{f}=7\cdot 10^{-8}$. The subsequent classes, from the second to the third, define increasingly stable devices. For them, we assume stability values of $s_{f}=\left\{2\cdot 10^{-9},\;7\cdot 10^{-12}\right\},$ respectively. These three $s_{f}$ values, $s_{f}=\left\{7\cdot 10^{-8},\;2\cdot 10^{-9},\;7\cdot 10^{-12}\right\},$ were calculated as the average values of the $s_{f}$ parameter for each class with the mantissa of the obtained result rounded to the unity. For example, for the first class, the average value of the stability parameter was $s_{f}=7.25\cdot 10^{-8}$. After rounding, $s_{f}=7\cdot 10^{-8}$ was assumed. The proposed classification of SDR platforms according to the frequency stability parameter is presented in Table 2. Additionally, based on Equation (4) and proposed values of $s_{f}$ we determine $\sigma_{f}$ for *f*_0_ = 1358 MHz. For example, in Table 2, for the first class, which represents all tested SDRs without the external oscillator FS725, the stability parameter is equal to the previously mentioned $s_{f}=7\cdot 10^{-8}$. According to Equation (4), after multiplying this value by *f*_0_ = 1358 MHz, the value of $\sigma_{f}=95.06\;\mathrm{Hz}$ is obtained.

The data presented in Table 1 show another critical property from the viewpoint of the estimation accuracy of the received signal carrier frequency. Namely, for the analyzed SDRs we obtain different average carrier frequencies of the recorded signals. In the absence of a reference signal from a highly stable external oscillator, the difference between the expected and measured values of the carrier frequency may range from several dozen Hz to several kHz. When using an external frequency standard these values are smaller, ranging from several Hz to fractional parts of Hz. For further simulation studies, we adopted three values that define a constant offset between the measured and expected values of the instantaneous frequency, i.e., $\mu_{f}=\left\{10.0,\;1.0,\;0.1\right\}\;\mathrm{Hz}$. The adopted values of $\mu_{f}$ are examples and do not represent any of the previously proposed device classes.

## 5. Simulation Studies

### 5.1. Scenarios and Assumptions

The simulation tests aimed to assess the impact of the SDR frequency instability on the SDF localization accuracy. The studies focused on determining the location error Δr, defined by Equation (3).

In our research, we assume that the location sensor was installed on the UAV. The UAV moves along a straight line at a constant speed of v=15 m/s [50] at an altitude of $h=-z_{0}=100\;m$ above the ground level [51]. The emitter continuously emits a harmonic signal at a constant and known carrier frequency $f_{0}=1358\;\mathrm{MHz}$. We conducted the research for two scenarios. The first was a short-range scenario in which the emitter was located 1 km from the SDF sensor route. The second was a long-range scenario. In this case, the emitter position was 10 km from the sensor trajectory. Additionally, we assumed that the instantaneous frequency of the received signal was a random variable with a normal distribution $N(\mu_{f},\sigma_{f})$, with a mean value $\mu_{f}$ and a standard deviation $\sigma_{f}$. Based on Section 4, we adopted the following values of $\mu_{f}=\left\{10.0,\;1.0,\;0.1\right\}\;\mathrm{Hz},$ and $\sigma_{f}=\left\{95.06,\;2.72,\;0.01\right\}\;\mathrm{Hz}$ for the simulation studies.

**Figure 4:**
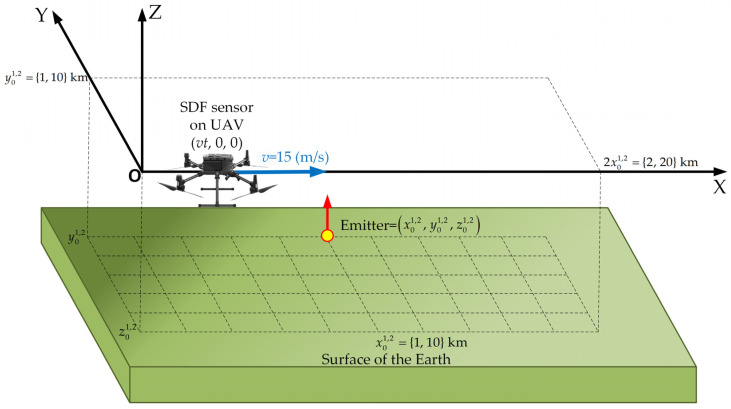
Spatial scenario for simulation studies.

In the simulation studies, the following additional assumptions were made:

- An illustrative spatial scenario, as shown in Figure 4.
- To define emitter and sensor positions, we used the local Cartesian coordinate system.
- The emitter was localized at a point $\left(x_{0}^{1},\;y_{0}^{1},\;z_{0}^{1}\right)=\left(1,\;1,\;-0.1\right)\left(\mathrm{km}\right)$ and $\left(x_{0}^{2},\;y_{0}^{2},\;z_{0}^{2}\right)=\left(10,\;10,\;-0.1\right)\left(\mathrm{km}\right)$ in the short- and long-range scenarios, respectively.
- The movement trajectory length of the sensor (i.e., UAV) was equal to $S_{1}=2\;\mathrm{km}$ and $S_{2}=20\;\mathrm{km}$ in the short- and long-range scenarios, respectively.
- The simplified SDF (i.e., 2D) version was used.
- The location sensor estimated the instantaneous DFS every 0.1 s.
- The coordinates of the emitter were estimated every 1 s based on 300 DFSs (i.e., the acquisition time of the received signal was equal to $t_{A}=30\;s$).
- A Monte Carlo simulation methodology was applied with *K* = 100 repetitions of statistical model realizations.

### 5.2. Results for Short-Range Scenario

The simulation studies were conducted for the analyzed scenario (see Figure 4) and the adopted assumptions. In the short-range scenario, the emitter is 1 km from the UAV (i.e., SDF sensor) trajectory.

**Figure 5:**
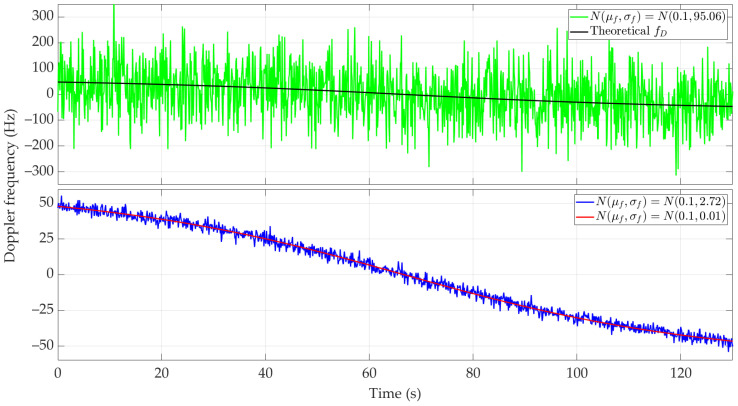
Example Doppler curves versus time for short-range scenario. Single realizations of random process for $\mu_{f}=0.1\;\mathrm{Hz}$ and three different $\sigma_{f}$.

**Figure 6:**
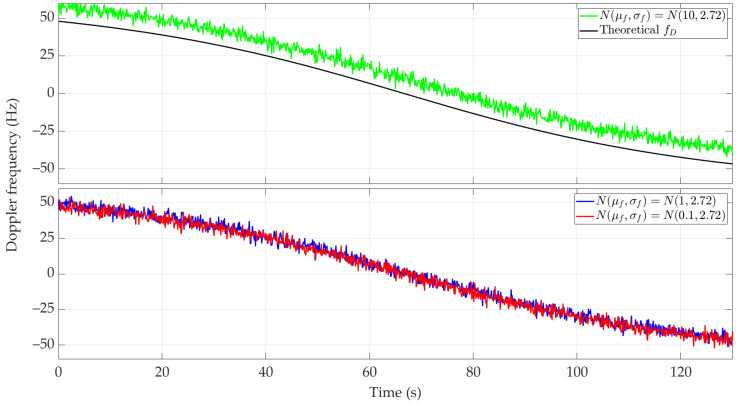
Example Doppler curves versus time for short-range scenario. Single realizations of random process for three selected $\mu_{f}$ and $\sigma_{f}=2.72\;\mathrm{Hz}$.

**Figure 7:**
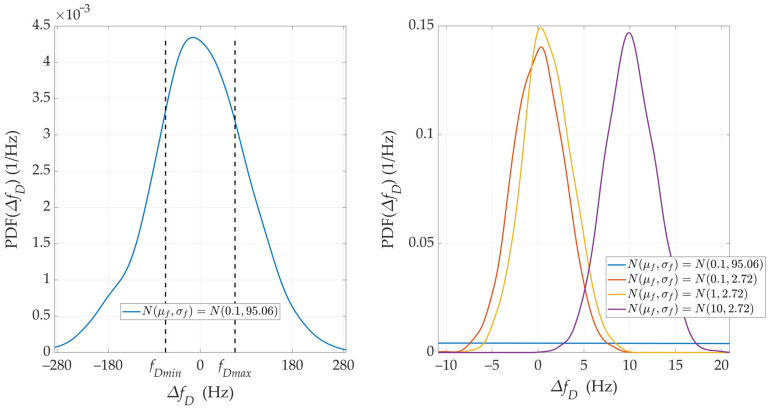
PDFs of DFS error for selected $\mu_{f}$ and $\sigma_{f}$.

Figure 5 and Figure 6 show the nature of the DFS changes versus time for a single realization of the random process. Figure 5 represents the variant for $\mu_{f}=0.1\;\mathrm{Hz}$ and three selected $\sigma_{f}$. Figure 6 depicts the case for three different $\mu_{f}$ and $\sigma_{f}=2.72\;\mathrm{Hz}$. These Doppler curves were created by randomizing instantaneous DFSs according to assumed distributions $N\left(\mu_{f},\;\sigma_{f}\right)$. In these figures, we also present the theoretical Doppler curve to highlight the effect of frequency instability on the generated Doppler curves. Figure 7 additionally shows probability density functions (PDFs) of the DFS error, which is defined as follows:

(5) $$\Delta f_{D}(t)={\tilde{f}}_{D}(t)-f_{D}(t).$$

The mean value $\mu_{f}$ introduces a constant DFS offset, which can be compensated in a relatively simple way, e.g., by measuring the frequency using a fixed sensor. On the other hand, the standard deviation $\sigma_{f}$ is a more significant parameter in the performed analysis. A large $\sigma_{f}$ causes the absolute DFS errors. For $\sigma_{f}=95.06\;\mathrm{Hz},$ the instantaneous DFSs often exceed the maximum DFS, which prevents the effective use of these results in the SDF method. This is shown below. In the analyzed case, $\sigma_{f}$ represents the receiver’s frequency instability. Hence, this parameter should be considered when choosing an SDR.

After *K* = 100 repetitions of statistical model realizations, the average localization error $\bar{\Delta r(}t)$ was determined according to the following formula:

(6) $$\bar{\Delta r(}t)=\frac{1}{K}\sum_{k=1}^{K}\Delta r_{k}(t),$$

where k=1,2,…,K, $\Delta r_{k}(t)$ is a location error defined by Equation (3) for the *k*th execution of a random process.

Additionally, to compare results for different $\mu_{f}$ and $\sigma_{f},$ we determined the mean value and standard deviation of the error location as follows:

(7) $$\mu_{\bar{\Delta r}}=E\left\{\bar{\Delta r}(t)\right\},$$

(8) $$\sigma_{\bar{\Delta r}}=\sqrt{E\left\{{\left(\bar{\Delta r}(t)-\mu_{\bar{\Delta r}}\right)}^{2}\right\}},$$

where E{·} is the expectation operator.

**Figure 8:**
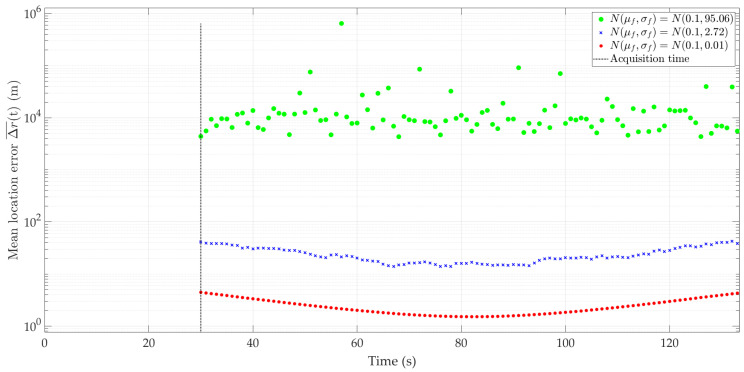
Average location error for short-range scenario,$\mu_{f}=0.1\;\mathrm{Hz},$ and three different $\sigma_{f}$.

**Figure 9:**
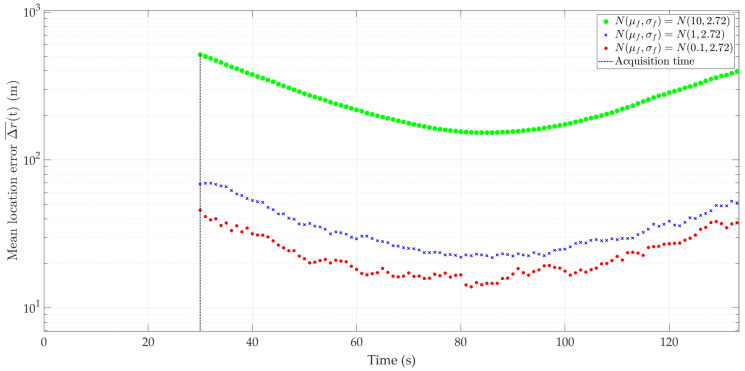
Average location error for short-range scenario, three selected $\mu_{f}$ and $\sigma_{f}=2.72\;\mathrm{Hz}$.

Figure 8 and Figure 9 show the average localization error $\bar{\Delta r(}t)$ for a constant $\mu_{f}$ with a variable $\sigma_{f}$ and variable $\mu_{f}$ with constant $\sigma_{f},$ and the parameter values as in Figure 5 and Figure 6, respectively. The influence of frequency stability parameters on the location error is presented in Table 3. We show the maximum and minimum values of the average localization error (see Equation (6)) for all assumed combinations of parameters $\mu_{f}$ and $\sigma_{f}$, and with a normal distribution $N\left(\mu_{f},\;\sigma_{f}\right)$. We also present the mean value and standard deviation (see Equations (7) and (8), respectively) of the average localization error.

As expected, higher $\sigma_{f}$ and $\mu_{f}$ values result in low localization accuracy. Considering the adopted device classes (see Table 2), the use of SDR without an external clock (i.e., class 1) results in localization errors exceeding the distance of the sensor to the localized object. Therefore, practical use of the SDF method is possible using classes 2 and 3. For large $\mu_{f}$ (e.g., $\mu_{f}=10$), the positioning errors obtained for these SDR classes are similar. For medium $\mu_{f}$ (e.g., $\mu_{f}=1$), using class 3 gives approximately a 20–30% reduction in localization error. Class 3 brings a significant improvement in localization accuracy for small $\mu_{f}$ (e.g., $\mu_{f}=0.1$). In this case, the position error can be reduced by 7–12 times compared to class 2.

### 5.3. Results for Long-Range Scenario

This scenario reflects the situation in which the emitter is located 10 km from the UAV (i.e., sensor) route. Greater distance between the emitter and the sensor results in a longer UAV flight time.

**Figure 10:**
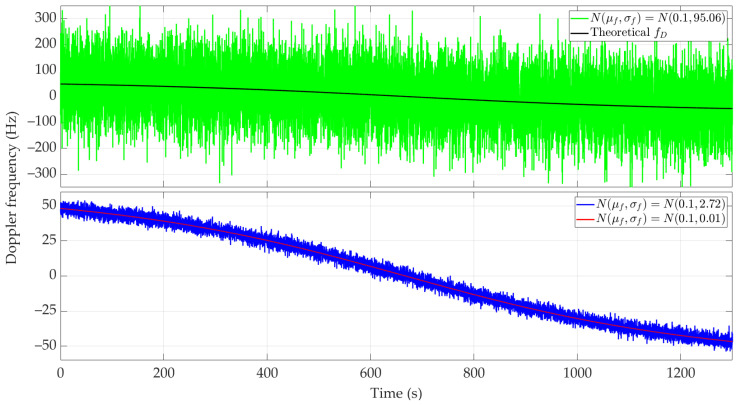
Example Doppler curves versus time for long-range scenario. Single realizations of random process for $\mu_{f}=0.1\;\mathrm{Hz}$ and three different $\sigma_{f}$.

**Figure 11:**
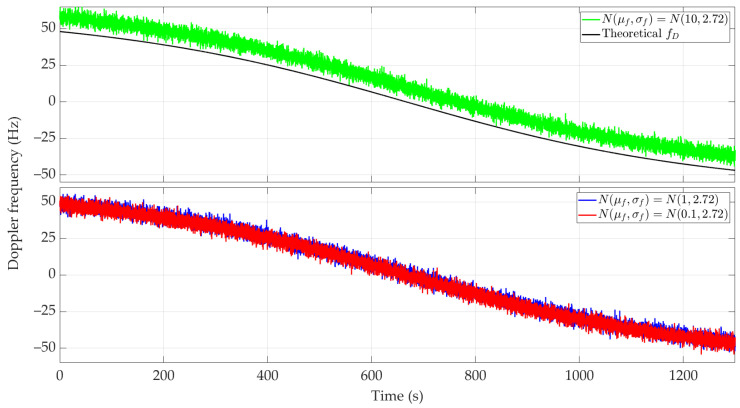
Example Doppler curves versus time for long-range scenario. Single realizations of random process for three selected $\mu_{f}$ and $\sigma_{f}=2.72\;\mathrm{Hz}$.

Figure 10 and Figure 11 show the Doppler curves versus time for a single realization of the random process. Figure 10 represents the variant for $\mu_{f}=0.1\;\mathrm{Hz}$ and three selected $\sigma_{f},$ Figure 11 depicts the case for three different $\mu_{f}$ and $\sigma_{f}=2.72\;\mathrm{Hz}$. These Doppler curves were created by randomizing instantaneous DFSs according to assumed distributions $N\left(\mu_{f},\;\sigma_{f}\right)$. In these figures, we also depict the theoretical Doppler curve to highlight the effect of frequency instability on the generated Doppler curves.

Since the same normal distribution parameters are considered for the short- and long-range scenarios, the corresponding Doppler curves are similar (see Figure 5 and Figure 6 and Figure 10 and Figure 11, respectively). For this reason, we do not present the PDFs for the long-range scenario. A significant change is a proportional increase in the length of the sensor route and the position coordinates of the located emitter relative to the sensor trajectory. This translates into a change in the recording time of the received signal and the number of estimated DFSs.

**Figure 12:**
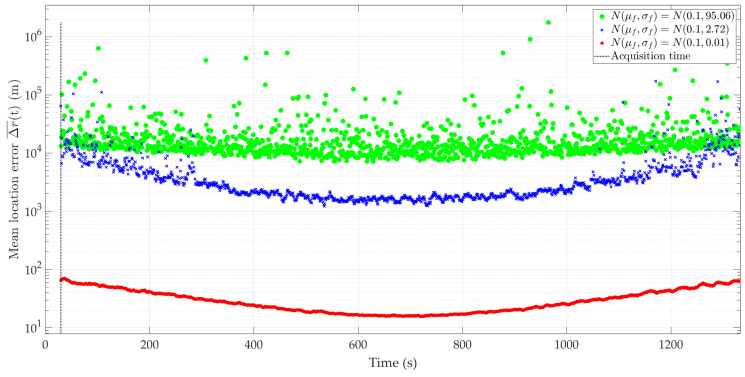
Average location error for long-range scenario, $\mu_{f}=0.1\;\mathrm{Hz},$ and three different $\sigma_{f}$.

**Figure 13:**
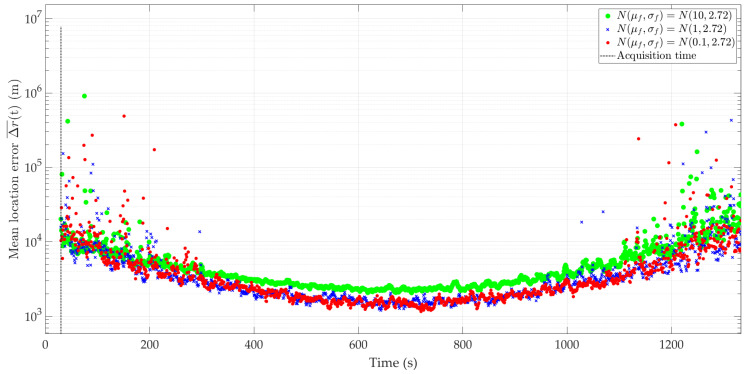
Average location error for long-range scenario, three selected $\mu_{f}$ and $\sigma_{f}=2.72\;\mathrm{Hz}$.

Similar to the previous scenarios, Figure 12 and Figure 13 illustrate the average localization error $\bar{\Delta r(}t)$ for a constant $\mu_{f}$ with a variable $\sigma_{f}$ and variable $\mu_{f}$ with a constant $\sigma_{f},$ and the parameter values as in Figure 8 and Figure 9, respectively. The simulation results are summarized in Table 4. We show the maximum and minimum values of the average localization error (see Equation (6)) for all assumed combinations of parameters $\mu_{f}$ and $\sigma_{f}$, and with a normal distribution $N\left(\mu_{f},\;\sigma_{f}\right)$. We also present the mean value and standard deviation (see Equations (7) and (8), respectively) of the average localization error.

In the long-range scenario, the localization errors obtained for classes 1 and 2 exclude using this type of device in the SDF method. As expected, the best accuracy was obtained for class 3. However, for a minor frequency offset case, its prior compensation is required.

## 6. Synthesis of Results

### 6.1. Scenario Comparision

The aim of the simulation studies was to evaluate the impact of SDR instability on emitter localization accuracy, which allows for selecting the SDR to be used as an element of the SDF sensor.

The specificity of DFS changes versus time for the analyzed acquisition window should be considered when comparing short- and long-range scenarios. In the studies, we assumed a constant value of this parameter for two scenarios. In the short-range scenario, the DFS changes in the acquisition window were greater than in the long-range scenario. This effect translates into larger localization errors for the long-range case. Therefore, when planning the mission, the appropriate direction of the sensor movement trajectory should be considered [27,33], or the value of the signal acquisition window should be adjusted [28].

On the other hand, the spatial relationship between the sensor movement trajectory and the located object position should be considered. Hence, to compare the two analyzed scenarios, the relative error is more appropriate than the absolute error. For this purpose, we define relative measures of the location error as follows:

(9) $$\delta_{\mu_{\bar{\Delta r}}}=\frac{\mu_{\bar{\Delta r}}}{\sqrt{y_{0}^{2}+z_{0}^{2}}}\cdot 100\%$$

(10) $$\delta_{\sigma_{\bar{\Delta r}}}=\frac{\sigma_{\bar{\Delta r}}}{\sqrt{y_{0}^{2}+z_{0}^{2}}}\cdot 100\%$$

where $\sqrt{y_{0}^{2}+z_{0}^{2}}$ is the shortest distance between the sensor and located emitter characterizing the so-called point of closest approach (PCA).

The calculated relative errors for SDR classes 2 and 3, for the short- and long-range scenarios, are contained in Table 5.

The presented results show that using good-class SDRs stabilized with an external rubidium or cesium clock allows for achieving high localization accuracy in methods based on measuring the received signal frequency. On the other hand, much larger errors in the long-range scenario indicate a significant impact of the chosen UAV motion trajectory and signal acquisition parameters on the SDF accuracy.

### 6.2. Comparison of Simulation and Empirical Results

The following comparison of the obtained simulation results with others available in the literature is based on the assumption of using the same localization method, i.e., SDF. In this case, frequency instability and its impact on the localization accuracy using the SDF method were analyzed only in [31]. Based on laboratory measurements, frequency stabilities were determined for three Keysight (Agilent) signal generators, i.e., N5172B, E8251A, and E4438C, at two frequencies, 1.449 and 1.629 GHz. A Rohde & Schwarz EB500 stabilized by a FS 725 rubidium standard was used as a measurement receiver. The obtained results (from 2.23 · 10^−10^ to 3.50 · 10^−10^) were the basis for evaluating the emitter location error using the SDF method. In the simulation studies, a long-range scenario was assumed, with four values each for frequency stability and acquisition time, equal to $\left\{1,2,4,8\right\}\cdot 10^{-10}$ and {30,60,90,120}(s), respectively. The obtained location errors ranged from 43 to 677 m depending on the acquisition time and frequency stability.

A similar approach based on simulation studies has been used for cooperative time-to-arrival (TOA) localization for UAV systems [52]. In this case, the authors analyzed the need to synchronize clocks to improve location accuracy. This solution is based on a synchronous two-way ranging process. The authors declare that the proposed approach outperforms existing methods and can achieve sub-nanosecond-level time synchronization and meter-level cooperative localization. However, it has not been confirmed by experiment.

The empirical tests conducted so far for the SDF method concerned only a very short-range scenario. Therefore, it difficult to compare the experimental results with the simulation studies presented in this paper for short- or long-range scenarios. Table 6 concludes the SDF location accuracy obtained in the empirical studies.

Empirical research was carried out for measurement scenarios located on a university campus using a car [30,53,54,55] or UAV [56]. In the first experiments [30,53,54], the obtained location errors were very small, which resulted from the fact that the emitter position was determined based on all DFSs measured along the measurement route, i.e., the emitter position was estimated based on the whole Doppler curve. In recent studies [55,56], the emitter position was determined based on DFS measured during the acquisition time window of approximately 30 s. The located signal source emitted a harmonic [53,54,55] or modulated signal, i.e., binary phase shift keying (BPSK), quadrature phase shift keying (QPSK), or 16 quadrature amplitude modulation (16 QAM) [30,56]. In this case of modulated signals, DFSs were determined based on the estimation of the carrier signal determined by raising the signal to the second or fourth power.

Atypical measurement results for the indoor scenario, conveyor belt, and ultra-high frequency (UHF) radio-frequency identification (RFID) technology are presented in [57]. In this case, the accuracy of the method was a single centimeter while the maximum distance between the transmitter and receiver was 2.4 m. Another difficulty in comparing the results of this experiment with simulation studies is the difficulty of finding information on the frequency stability of RFID devices.

Comparing the location errors obtained in simulation studies with empirical ones is difficult, primarily due to the different nature of the used scenarios. We should classify the empirical test scenarios as very short range, while in the simulations, we defined short- to long-range scenarios. For comparison, the maximum distances between the emitter and receiver were 56 ÷ 395, 1418, and 14,142 m for the very short-, short-, and long-range scenarios, respectively. However, it should be noted that considering vehicle to emitter distances (see Equation (9)), the relative location errors may be approximate for class 3 SDR devices.

Generally, as the distance increases, the measurement time increases, which shortens the range of DFS variability in the data acquisition time. As mentioned above, the first empirical studies [30,53,54] used the entire range of DFS variability, which significantly improved the accuracy of the localization method. This is not possible when the measurement route is located, i.e., the UAV flight takes place, at a considerable distance from the located emitter.

The impact assessment of frequency instability of selected SDRs is aimed at selecting an appropriate platform for the developed Doppler-based localization sensor. On the other hand, the conducted simulation studies were intended to initially assess the accuracy of the SDF method for short- and long-range scenarios. We plan to carry out empirical studies in real conditions for the two analyzed scenarios in the next stage of the UAV-COMINT project. In this case, we will use the developed SDF-based localization sensor.

### 6.3. SDR Comparison

Considering the target operating frequency range of the location sensor, weight limitations, and dimension limitations resulting from the capabilities of the UAV used, we compared SDR platforms in terms of their operating frequency range and physical dimensions. The operating frequency range and bandwidth were read from the datasheets [41,42,43,44,45,46,47,48]. The platform dimensions were measured as the maximum size of the SDR housing without the length of protruding connectors. The obtained results are summarized in Table 7.

Based on the research assumptions from Section 5.1., analogous simulations were performed for the parameters $\mu_{f}$ and $\sigma_{f}$ obtained from that section with the rubidium frequency standards of Table 1. This study aimed to compare the possibilities of using specific SDR platforms in the location sensor. The obtained results are summarized in Table 8.

Based on the size list and simulation results presented in Table 6 and Table 7, respectively, a comprehensive comparison of available SDRs can be made in terms of their use in the location sensor. 

Considering the weight and size of the platforms, the bladeRF, B200mini-i, and ADALM-PLUTO seem to be the best solutions. The remaining SDRs would require dismantling the dedicated case and trying to reduce the size by designing your own solutions. It can be seen that the B200mini-i and bladeRF platforms offer the most extensive range of operating frequencies in the presented configurations. ADALM-PLUTO has a slightly smaller range. However, when comparing the stability parameters of the ADALM-PLUTO with other solutions, it is the weakest. So, we may conclude that the error obtained during the simulation disqualifies using this platform in a location sensor. 

To sum up, the best choice of an SDR for use in a location sensor mounted on a UAV seems to be the B200mini-i and bladeRF 2.0 micro xA4. They have small dimensions, a low weight, and satisfactory stability parameters. We recommend their selection for subsequent empirical research.

### 6.4. Small-Size Frequency Oscillator Overview

In Section 4, frequency stability tests of the low-cost SDRs were made for Rubidium Frequency Standard FS725 [59]. Due to its significant size, weight, and power consumption, it cannot remain in the location sensor that is to be ultimately mounted on the UAV. For this reason, we conducted an overview of available external reference clocks, summarized in Table 9. It aimed to select the most appropriate oscillator.

The simplest solution for the practical implementation of a local generator of reference clock signals is to use a stable thermally stabilized quartz generator oven-controlled quartz oscillator (OCXO) synchronized with the signal obtained from a global navigation satellite system (GNSS) receiver. However, the problem is achieving high accuracy and stability when the GNSS signal is unavailable (e.g., in a street canyon) or strong interference (e.g., GNSS jamming) occurs preventing the use of this approach. Local generators using atomic (i.e., rubidium or cesium) resonators are the solution in these cases.

Table 9 presents selected atomic clock models currently available on the market, dedicated to applications as local sources of reference signals, e.g., mounted on UAVs. A comprehensive examination of most of the listed clocks was carried out in [71]. The authors included a comparison of performance versus size and power for current external clocks and compared early prototypes of next-generation frequency standards to current product trends. It has been mentioned that applications requiring extremely low power (i.e., on the order of less than 1 W) to achieve their mission currently should utilize a chip-scale atomic clock (CSAC).

For operational reasons, the synchronization time is also crucial. The synchronization time of clocks 1–6 from Table 9 exceeds 15 min. Clocks 7–10 need about 8 min. However, Microchip SA.45s CSAC [70] (i.e., the 11th position in Table 9) synchronizes even below 130 s. 

**Figure 14:**
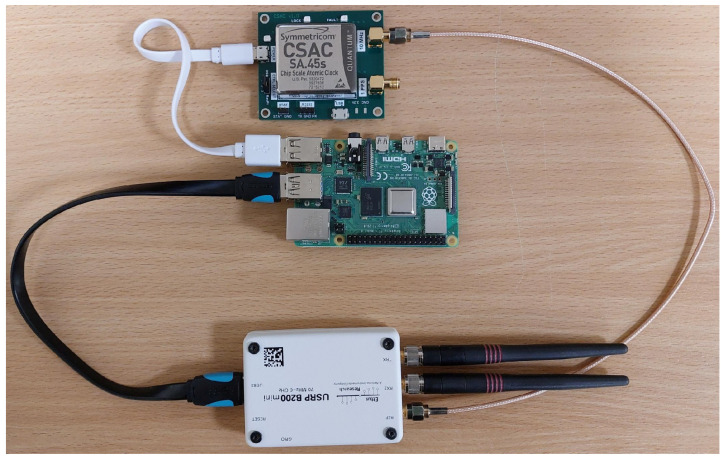
Hardware structure for a location sensor consisting of SA.45s CSAC, Raspberry Pi 4 model B, and USRP B200mini-i.

**Figure 15:**
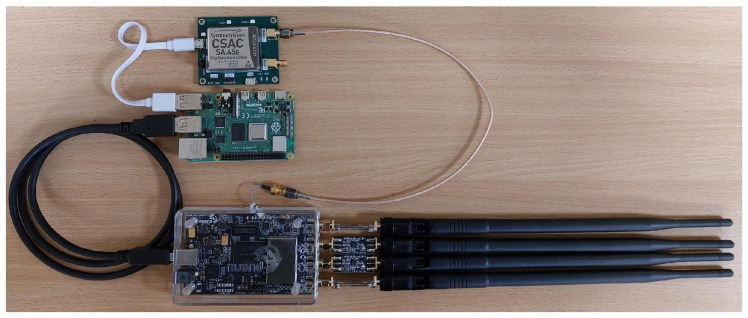
Hardware structure for a location sensor consisting of SA.45s CSAC, Raspberry Pi 4 model B, and bladeRF 2.0 micro xA4.

Considering the above parameters, the best solution in mobile applications seems to be the use of SA.45s CSAC. This generator, thanks to its unconventional structure, presented at the top of Figure 14 and Figure 15, and the use of a miniature cesium resonator [70], is characterized by extremely low power consumption. It is the only atomic standard that does not use thermal stabilization (oven-controlled) techniques. The device focuses on reducing power consumption, and the electronic board is placed in a hermetic, vacuum metal housing. The practical lack of heat exchange with the environment and the low power losses of the electronic components placed inside the case mean that the thermal compensation technique is sufficient for proper operation.

The SA.45s generator can also be programmed to operate in very low power mode. In this mode, the CSAC (cesium laser resonator) physics package is turned off and the atomic clock turns into a free-running temperature-compensated crystal oscillator (TCXO). The physics package is then periodically turned back on, and after it warms up (<130 s) the TCXO generator synchronization process with the signal from the cesium resonator is performed again. This operating mode allows an average power consumption level well below 50 mW. CSAC SA.45s also has a military version called SA.65s.

To summarize the SDR comparison carried out in Section 6.3 and the overview of the small-size frequency oscillators presented in Section 6.4, we decided to propose two possible hardware configurations of the location sensor characterized by a small size, low weight, and low power consumption, which are visible in Figure 14 and Figure 15. Both configurations include (mentioned successively from the top of the drawing) the SA.45s CSAC oscillator and the Raspberry Pi 4 model B microcomputer. However, they differ in the SDR used. In Figure 14, we see the USRP B200mini-i. Figure 15 presents the configuration with the slightly larger bladeRF 2.0 micro xA4 system.

## 7. Conclusions

In this paper, we presented the effect of SDR platforms’ frequency instability on the SDF localization accuracy. The evaluation of available low-cost SDRs in terms of their frequency stability allowed us to select an appropriate platform for building the Doppler-based location sensor. Our analysis was based on frequency stability measurements carried out in two variants, i.e., using an internal and external clock for six selected SDRs. In the second case, we used Rubidium Frequency Standard FS725. Based on these measurement results, we defined three classes of devices with respect to their frequency stability. The proposed classification and representative parameter values were the basis for the simulation studies. Simulations were carried out for two spatial scenarios, i.e., short- and long-range, in which absolute and relative location errors were determined. This was the basis for the assessment of individual device classes as well as selected SDR platforms. We compared the simulation results with available empirical test results for SDF. Comparing absolute location errors is difficult because the experimental study scenario should be classified as very short range or indoor. However, relative errors may be approximate for a class 3 SDR device.

The obtained results allowed us to clearly state that SDF localization absolutely requires connecting an external clock to the SDR platform. To reduce positioning errors, it is necessary to use an SDR with low frequency instability. Moreover, frequency offset compensation can significantly improve SDF accuracy, especially when its values are significant. Additionally, appropriate mission planning, which should provide for the UAV flight trajectory selection concerning the emitter position, is a crucial factor that should be considered in the SDF method. 

In the paper, we additionally provide an overview of small-size frequency oscillators. In future research, we want to check the frequency stability of the B200mini-i and bladeRF 2.0 micro xA4 with SA.45s as an external clock. The proposed configurations of the SDF sensor characterize good frequency stability, small size and weight, and low power consumption appropriate for UAV application. Next, we plan to place the location sensor on the UAV and conduct empirical tests of the SDF accuracy in a real environment.

## Figures and Tables

**Table 1:** Frequency stability results for various SDR platforms with *f*_0_ = 1358 MHz.

SDR Platform	Without Rubidium Oscillator			With Rubidium Oscillator		
	$\mu_{f}$ (Hz)	$\sigma_{f}$ (Hz)	$s_{f}$ (–)	$\mu_{f}$ (Hz)	$\sigma_{f}$ (Hz)	$s_{f}$ (–)
ADALM-PLUTO	16,757.1	285.9	2.11∙10^−7^	−55.155	5.113	3.77∙10^−9^
B200mini-i	1436.7	5.5	4.61∙10^−9^	−0.004	1.287	9.48∙10^−10^
N210 + WBX	1010.3	179.9	1.21∙10^−7^	0.011	0.014	1.01∙10^−11^
NI–2950R	928.3	13.9	9.62∙10^−9^	−0.011	0.012	8.71∙10^−12^
bladeRF 2.0 micro xA4	−37.5	34.7	2.40∙10^−8^	−0.201	0.010	7.12∙10^−12^
N210 + RFX1200	1180.1	78.6	6.08∙10^−8^	0.012	0.008	5.68∙10^−12^
NI–2930	93.0	96.3	7.68∙10^−8^	0.009	0.005	3.91∙10^−12^

SDR device classes: yellow—1st class, blue—2nd class, green—3rd class.

**Table 2:** Proposed classification of SDR platforms.

Device Class	SDR Platform		$s_{f}$ (–)	$\sigma_{f}$ (Hz) for *f*_0_ = 1358 MHz
1	All tested SDRs without the external oscillator FS725		$7\cdot 10^{-8}$	95.06
2	ADALM-PLUTO B200mini-i	with FS725	$2\cdot 10^{-9}$	2.72
3	N210 + WBX NI–2950R bladeRF 2.0 micro xA4 N210 + RFX1200 NI–2930	with FS725	$7\cdot 10^{-12}$	0.01

SDR device classes: yellow—1st class, blue—2nd class, green—3rd class.

**Table 3:** Influence of frequency stability parameters on location error for short-range scenario.

Frequency Stability Parameters (Hz)	Location Error (m)			
$N\left(\mu_{f},\;\sigma_{f}\right)$	$\min\left(\bar{\Delta r}(t)\right)$	$\max\left(\bar{\Delta r}(t)\right)$	$\mu_{\bar{\Delta r}}$	$\sigma_{\bar{\Delta r}}$
*N* (10.0, 95.06)	3149	119,810	14,980	18,213
*N* (10.0, 2.72)	153	511	251	94
*N* (10.0, 0.01)	151	474	246	88
*N* (1.0, 95.06)	3384	258,415	16,523	28,917
*N* (1.0, 2.72)	21	75	34	12
*N* (1.0, 0.01)	15	44	25	9
*N* (0.1, 95.06)	3329	97,601	15,013	16,527
*N* (0.1, 2.72)	14	45	24	8
*N* (0.1, 0.01)	2	4	2	1

SDR device classes: yellow—1st class, blue—2nd class, green—3rd class.

**Table 4:** Impact of frequency stability parameters on location error for long-range scenario.

Frequency Stability Parameters (Hz)	Location Error (m)			
$N\left(\mu_{f},\;\sigma_{f}\right)$	$\min\left(\bar{\Delta r}(t)\right)$	$\max\left(\bar{\Delta r}(t)\right)$	$\mu_{\bar{\Delta r}}$	$\sigma_{\bar{\Delta r}}$
*N* (10.0, 95.06)	6767	3,576,176	26,340	128,434
*N* (10.0, 2.72)	2026	167,774	6391	10,594
*N* (10.0, 0.01)	1470	6685	2988	1421
*N* (1.0, 95.06)	6693	2,173,297	23,394	89,370
*N* (1.0, 2.72)	1187	162,559	4920	8730
*N* (1.0, 0.01)	147	637	299	142
*N* (0.1, 95.06)	6575	603,692	19,695	29,817
*N* (0.1, 2.72)	1233	227,920	5311	11,285
*N* (0.1, 0.01)	16	68	31	14

SDR device classes: yellow—1st class, blue—2nd class, green—3rd class.

**Table 5:** Relative errors for short- and long-range scenarios.

Frequency Stability Parameters (Hz)	Short-Range Scenario		Long-Range Scenario	
$N\left(\mu_{f},\;\sigma_{f}\right)$	$\delta_{\mu_{\bar{\Delta r}}}$ (%)	$\delta_{\sigma_{\bar{\Delta r}}}$ (%)	$\delta_{\mu_{\bar{\Delta r}}}$ (%)	$\delta_{\sigma_{\bar{\Delta r}}}$ (%)
*N* (1.0, 2.72)	3.4	1.2	49.2	87.3
*N* (1.0, 0.01)	2.5	0.9	3.0	1.4
*N* (0.1, 2.72)	2.4	0.8	53.1	112.8
*N* (0.1, 0.01)	0.2	0.1	0.3	0.1

SDR device classes: blue—2nd class, green—3rd class.

**Table 6:** Localization accuracy of radio emitters using SDF method based on empirical research.

Works (Year)	Vehicle (Speed)	Emitter/Signal Generator (Emitted Signal Type; Carrier Frequency)	Localization Sensor/Receiver	Location Error	Emitter–Receiver Maximum Distance
[53] (2008)	Car (v=10 m/s)	Hammeg HM81384-3 stabilized by FS 725 rubidium standard (transmitted signal: harmonic; $f_{0}=900\;\mathrm{MHz}$)	Rohde & Schwarz ESMC-R1 stabilized by FS 725 rubidium standard	<1.1 m	56 m
[54] (2011)	Car (v=10 m/s)	Hammeg HM81384-3 stabilized by FS 725 rubidium standard (transmitted signal: harmonic; $f_{0}=900\;\mathrm{MHz}$)	Rohde & Schwarz ESMC-R1 stabilized by FS 725 rubidium standard	<2.3 m	65 m
[30] (2015)	Car (v=10 m/s)	Hammeg HM81384-3 stabilized by FS 725 rubidium standard (transmitted signal: BPSK and QPSK; $f_{0}=1.832\;\mathrm{GHz}$)	Rohde & Schwarz EM550 stabilized by FS 725 rubidium standard	2–9 m	80 m
[55] (2019)	Car (v=10 m/s)	Rohde & Schwarz SMIQ02 stabilized by FS 725 rubidium standard (transmitted signal: harmonic; $f_{0}=1.832\;\mathrm{GHz}$)	Ettus Research USRP B200mini-i	2–37 m	148 m
[56] (2023)	UAV (v=15 m/s)	Ettus Research USRP B200mini-i stabilized by FS 725 rubidium standard (transmitted signal: BPSK, QPSK, and 16 QAM; $f_{0}=2.3\;\mathrm{GHz}$)	Ettus Research USRP B200mini-i	2–17 m	395 m
[57] (2015)	Conveyor belt (v=0.4m/s)	RFID tag (transmitted signal: standard UHF RFID; $f_{0}=924\;\mathrm{MHz}$)	Impinj Speedway Revolution R420 RFID reader	0.052 m	2.4 m

**Table 7:** Comparison of operating frequency range and physical dimensions of available SDRs.

No.	SDR Platform	Frequency Range (MHz)		Bandwidth (MHz)	Dimensions * (mm)	Weight (g)
		Tx	Rx			
1	ADALM-PLUTO	70–3800 **	70–3800 **	20 **	78 × 117 × 23	116
2	B200mini-i	70–6000	70–6000	56	55 × 79 × 16	108
3	N210 + WBX	50–2200	50–2200	40	160 × 204 × 48	1160
4	NI–2950R	50–2200	50–2200	120	218 × 267 × 39	1787
5	bladeRF 2.0 micro xA4	47–6000	70–6000	56	72 × 110 × 24	112
6	N210 + RFX1200	1150–1450	1150–1450	40	160 × 204 × 48	1160
7	NI–2930	50–2200	50–2200	40	160 × 204 × 48	1218

* width × depth × height, ** possible to perform a quick hack that changes the frequency range and bandwidth from 325–3800 MHz and 20 MHz up to 70 MHz to 6000 MHz and 56 MHz bandwidth [58].

**Table 8:** Influence of frequency stability parameters on location error for all available SDR platforms.

No.	SDR Platform	$\mu_{f}$ (Hz)	$\sigma_{f}$ (Hz)	$\mathrm{Location}\;\mathrm{Error}\;\mu_{\bar{\Delta r}}\pm \sigma_{\bar{\Delta r}}$ (m)	
				Short-Range Scenario	Long-Range Scenario
1	ADALM-PLUTO	−55.155	5.113	± 1897.8	± 51,347.2
2	B200mini-i	−0.004	1.287	± 3.4	± 668.6
3	N210 + WBX	0.011	0.014	± 0.1	± 6.4
4	NI–2950R	−0.011	0.012	± 0.1	± 5.2
5	bladeRF 2.0 micro xA4	−0.201	0.010	± 1.7	± 28.6
6	N210 + RFX1200	0.012	0.008	± 0.1	± 3.7
7	NI–2930	0.009	0.005	± 0.1	± 2.5

**Table 9:** Selected currently available reference atomic clocks.

No.	Model	Type	ADEV @ 100 s	Power Consumption (W)	Dimensions * (mm)	Weight (g)
1	Stanford Research Systems PRS-10 [60]	Rb	$2\cdot 10^{-12}$	53	76.2 × 50.8 × 101.6	600
2	AccuBeat AR133A [61]	Rb	$5\cdot 10^{-12}$	18	77.0 × 77.0 × 25.4	295
3	Microsemi SA.31m, SA.33m, SA.35m [62]	Rb	$8\cdot 10^{-12}$	14	50.8 × 50.8 × 18.3	85
4	Morion RFS-M102 [63]	Rb	$3\cdot 10^{-12}$	18	51.0 × 51.0 × 25.0	-
5	Safran LPFRS [64]	Rb		20	76.0 × 77.0 × 36.5	290
6	IQD IQRB-1 [65]	Rb	$6\cdot 10^{-12}$	20	50.8 × 50.8 × 25.0	-
7	AccuBeat NAC1 [66]	Rb	$2\cdot 10^{-11}$	2.4	41.1 × 35.8 × 22.0	75
8	Microchip MAC-SA5X (SA53, SA55) [67]	Rb	$3\cdot 10^{-12}$	8	50.8 × 50.8 × 18.3	100
9	Quartzlock E10-CPT [68]	Rb	$2\cdot 10^{-11}$	5.2	45.0 × 36.0 × 15.0	45
10	Safran mRO-50 Ruggedized [69]	Rb	$6\cdot 10^{-12}$	1.5	50.8 × 50.8 × 20.0	80
11	Microchip SA.45s CSAC [70]	Cs	$3\cdot 10^{-11}$	0.14	35.3 × 40.6 × 11.4	35

* width × depth × height.

## Data Availability

The data presented in this study are available on request from the corresponding author.
